# Live-cell Microscopy and Fluorescence-based Measurement of Luminal pH in Intracellular Organelles

**DOI:** 10.3389/fcell.2017.00071

**Published:** 2017-08-21

**Authors:** Li Ma, Qing Ouyang, Gordon C. Werthmann, Heather M. Thompson, Eric M. Morrow

**Affiliations:** ^1^Department of Molecular Biology, Cell Biology and Biochemistry, Brown University Providence, RI, United States; ^2^Brown Institute for Brain Science, Brown University Providence, RI, United States; ^3^Hassenfeld Child Health Innovation Institute, Brown University Providence, RI, United States

**Keywords:** endosome, fluorescence, Golgi, lysosome, organelle, pHluorin, pH measurement, ratiometric

## Abstract

Luminal pH is an important functional feature of intracellular organelles. Acidification of the lumen of organelles such as endosomes, lysosomes, and the Golgi apparatus plays a critical role in fundamental cellular processes. As such, measurement of the luminal pH of these organelles has relevance to both basic research and translational research. At the same time, accurate measurement of intraorganellar pH in living cells can be challenging and may be a limiting hurdle for research in some areas. Here, we describe three powerful methods to measure rigorously the luminal pH of different intracellular organelles, focusing on endosomes, lysosomes, and the Golgi apparatus. The described methods are based on live imaging of pH-sensitive fluorescent probes and include: (1) A protocol based on quantitative, ratiometric measurement of endocytosis of pH-sensitive and pH-insensitive fluorescent conjugates of transferrin; (2) A protocol for the use of proteins tagged with a ratiometric variant of the pH-sensitive intrinsically fluorescent protein pHluorin; and (3) A protocol using the fluorescent dye LysoSensor™. We describe necessary reagents, key procedures, and methods and equipment for data acquisition and analysis. Examples of implementation of the protocols are provided for cultured cells derived from a cancer cell line and for primary cultures of mouse hippocampal neurons. In addition, we present strengths and weaknesses of the different described intraorganellar pH measurement methods. These protocols are likely to be of benefit to many researchers, from basic scientists to those conducting translational research with a focus on diseases in patient-derived cells.

## Introduction

Acidification of the lumen of intracellular organelles (such as endosomes, lysosomes, and the Golgi apparatus) plays a critical role in fundamental cellular processes. These processes include, for example, vesicle trafficking and fusion, receptor–ligand interactions and related signaling, lysosomal degradation and autophagy, and protein and lipid post-translational modification such as glycosylation (Huotari and Helenius, 2011). Maintenance of the luminal pH of intracellular organelles within a narrow range appropriate for a given organelle and cell type involves tight regulation of channels, transporters, exchangers, and pumps to ensure appropriate balance of cations and anions (Casey et al., 2010). As indication of the importance of this process, intraorganellar acidification may be defective in a variety of diseases, including lysosomal storage diseases (Bach et al., 1999; Holopainen et al., 2001; Fukuda et al., 2006), developmental disorders (Condon et al., 2013; Ouyang et al., 2013), and potentially neurodegenerative diseases such as Alzheimer's disease (Colacurcio and Nixon, 2016).

Along the endocytic pathway, vesicles formed at the plasma membrane—where the external milieu has a pH of ~7.4—are initially trafficked to, or fuse to generate, early endosomes (pH ~6.0–~6.5) (Murphy et al., 1984). From here, vesicles may traffic to recycling endosomes, which are generally more alkaline (pH of ~6.4–~6.5) (Yamashiro et al., 1984). Alternatively, early endosomes, together with any remaining cargo contents, may mature into late endosomes, which are generally more acidic (pH ~5.5–~6.0). Continuing down the protein degradation pathway and acidifying even further, the late endosomes eventually fuse with lysosomes (pH ~4.5–~5.5). Similar to the endocytic pathway, proteins and lipids transitioning along the biosynthetic pathway—from the endoplasmic reticulum (ER) to, and through, the Golgi apparatus—progress from a luminal environment of relatively neutral pH (ER, pH ~7.2) to luminal environments of increasingly greater acidity (cis- to trans-Golgi, pH ~6.7–~6.0). Furthermore, some post-Golgi carriers are involved in the delivery of lysosomal enzymes, and thus these vesicles encounter the acidic environments of endosomes and lysosomes. Of note, the exact pH-values of the indicated intracellular organelles vary depending on cell type and the specific function or cargo content of an organelle or vesicle (Sipe et al., 1991; Rybak and Murphy, 1998). Nevertheless, the existence of organelles or organelle compartments of a progressively more acidic environment in moving inward along the endocytic pathway or outward along the secretory pathway can be considered a general trend (for reviews, see: Weisz, 2003; Maxfield and McGraw, 2004; Huotari and Helenius, 2011).

A luminal acidic environment is an important functional component of both the endocytic and secretory pathways. Indeed, the ability of alkalinizing weak bases (“lysosomotropic agents”), K^+^/H^+^ and Na^+^/H^+^ ionophores, and inhibitors of the vacuolar ATPase (v-ATPase) to alter cellular activities—such as receptor recycling, protein and lipid sorting or degradation, and luminal post-translational modification of proteins—by modifying the luminal pH is well-established (Reijngoud et al., 1976; Ohkuma and Poole, 1978; Maxfield, 1982; Tartakoff, 1983; Yamashiro et al., 1983; Bowman et al., 1988). Furthermore, these pH-altering drugs are a commonly used tool in the field of vesicle trafficking, although caution must be exhibited in interpreting the results when using such reagents (Weisz, 2003). From a disease-related perspective, protein mutations or genetic nulls leading to a cellular phenotype of altered organellar pH have been associated with defects in vesicle trafficking, receptor signaling, lysosomal degradation, or Golgi-mediated glycosylation, with the affected process depending on the mutation at hand (for examples, see: Bach et al., 1999; Holopainen et al., 2001; Fukuda et al., 2006; Condon et al., 2013; Ouyang et al., 2013; Colacurcio and Nixon, 2016).

For more than 30 years, investigators have been using fluorescent conjugates of growth factor receptor ligands to measure the pH of endocytic organelles and to determine the kinetics of organellar acidification along the endocytic pathway (Murphy et al., 1982, 1984; Tycko and Maxfield, 1982; van Renswoude et al., 1982; Sipe and Murphy, 1987). As such, it has long been appreciated that pH is an important functional feature of intracellular membranous organelles involved in endocytic vesicle trafficking and that its measurement may have relevance to both basic research and translational research. Here, we describe three methods to measure rigorously the luminal pH of different intracellular organelles, focusing on endosomes, lysosomes, and the Golgi apparatus. The described methods are based on live imaging of pH-sensitive fluorescent probes and include: (1) A protocol based on quantitative, ratiometric measurement of endocytosis of pH-sensitive, and pH-insensitive fluorescent conjugates of transferrin; (2) A protocol for the use of proteins tagged with a ratiometric variant of the pH-sensitive intrinsically fluorescent protein pHluorin; and (3) A protocol using the fluorescent dye LysoSensor™. Each method has different strengths and weaknesses, as discussed below. As such, use of different methods to corroborate findings augments confidence in interpretation of results.

## Materials and equipment

### Cell culture

To provide examples of implementation of the described protocols for measuring organellar pH, we have used: (1) a purchased line of cultured cells, namely, HAP1 cells (Horizon Discovery, Vienna, Austria) and (2) mouse primary hippocampal neurons dissected from post-natal day 0 (P0) to P1 mice. However, it is noted that the described methods can be applied to a wide variety of cell types. For HAP1 cell cultures, cells were grown in Iscove's Modified Dulbecco's Medium (IMDM) supplemented with 10% fetal bovine serum (FBS) and 1% penicillin–streptomycin. Cell cultures were maintained at 37°C in a humidified atmosphere of 95% air and 5% CO_2_. Cell culture medium and reagents used for HAP1 cells were obtained from ThermoFisher Scientific. For primary cultures of mouse hippocampal neurons, hippocampi were dissected from P0 to P1 mice, dissociated with papain (20 units/mL) in Earle's Balanced Salt Solution (EBSS) with bicarbonate at 37°C for 30 min, and triturated with a 1 mL pipette. Hippocampal neurons were plated on 35 mm glass bottom dishes (MatTek, Ashland, MA) pre-coated with 1 mg/mL poly-D-lysine at a cell density of 1.3 × 10^5^ cells/mL in Neurobasal®-A medium supplemented with 2% B-27®, 1% GlutaMAX™, and 1% penicillin–streptomycin. Primary neuronal cultures were maintained at 37°C in a humidified atmosphere of 95% air and 5% CO_2_. Cell culture medium and reagents used for primary neuronal cultures were obtained from Worthington Biochemical Corporation (Lakewood, NJ). All experiments involving mice were carried out in accordance with the National Institutes of Health *Guide for the Care and Use of Laboratory Animals* (National Research Council of the National Academies, 2011). The protocol was approved by the Brown University Institutional Animal Care and Use Committee.

### pH calibration curve buffers

For each protocol, a pH calibration curve needs to be generated in parallel with obtaining experimental data. Additionally, careful consideration should be given to ensuring that calibration curves obtained under similar conditions and using the same types of probes are consistent and exhibit a dynamic range appropriate for making accurate, reliable estimates of organellar pH^1^. For experiments for which results are provided herein, the pH calibration curve was generated as described previously (Xinhan et al., 2011; Ouyang et al., 2013) and as outlined below. The buffers for generating the pH calibration curve contain: 125 mM KCl, 25 mM NaCl, 10 μM monensin, and 25 mM *N*-[2-hydroxyethyl]-piperazine-*N*-[2-ethanesulfonic acid] (HEPES, pH 7.5 or 7.0) or 25 mM 2-[*N*-morpholino] ethanesulfonic acid (MES, pH 6.5, 6.0, 5.5, 5.0, 4.5, 4.0, or 3.5). Each buffer solution is adjusted to the appropriate final pH using 1 N NaOH or 1 N HCl. See Table 1 for stock solutions and respective volumes for generating 50 mL aliquots of the pH calibration curve buffers.

**Table 1:** Recipes for preparing pH calibration curve buffers.

	**1 M KCl**	**5 M NaCl**	**72 mM Monensin**	**0.5 M HEPES**	**0.5 M MES**	1 N NaOH^*^	1 N HCl^*^	**H_2_O**
Buffer of pH 7.5	6.25 mL	0.25 mL	6.9 μL	2.5 mL	–	^*^	^*^	to 50 mL
Buffer of pH 7.0	6.25 mL	0.25 mL	6.9 μL	2.5 mL	–	^*^	^*^	to 50 mL
Buffer of pH 6.5	6.25 mL	0.25 mL	6.9 μL	–	2.5 mL	^*^	^*^	to 50 mL
Buffer of pH 6.0	6.25 mL	0.25 mL	6.9 μL	–	2.5 mL	^*^	^*^	to 50 mL
Buffer of pH 5.5	6.25 mL	0.25 mL	6.9 μL	–	2.5 mL	^*^	^*^	to 50 mL
Buffer of pH 5.0	6.25 mL	0.25 mL	6.9 μL	–	2.5 mL	^*^	^*^	to 50 mL
Buffer of pH 4.5	6.25 mL	0.25 mL	6.9 μL	–	2.5 mL	^*^	^*^	to 50 mL
Buffer of pH 4.0	6.25 mL	0.25 mL	6.9 μL	–	2.5 mL	^*^	^*^	to 50 mL
Buffer of pH 3.5	6.25 mL	0.25 mL	6.9 μL	–	2.5 mL	^*^	^*^	to 50 mL
Final concentration	125 mM KCl	25 mM NaCl	10 μM Monensin	25 mM HEPES	25 mM MES	–	–	–

* *Each buffer solution is adjusted to the appropriate final pH using 1 N NaOH or 1 N HCl*.

### Fluorescent transferrin conjugates

Fluorescein isothiocyanate (FITC)-conjugated transferrin (FITC-Tfn) and Alexa Fluor® 546-conjugated transferrin (Alexa Fluor® 546-Tfn) were from ThermoFisher Scientific. Of note, FITC-transferrin specifically needs to be used as opposed to Alexa Fluor® 488-transferrin, as FITC is pH-sensitive whereas Alexa Fluor® 488 is pH-stable across the pH range of interest. To this end, for the described protocol, FITC-Tfn acts as a pH sensor, whereas the Alexa Fluor® 546-Tfn acts as an internal standard for assessment of endocytic uptake and organellar localization. The reagent powders were diluted in Milli-Q H_2_O to stock solutions of 5 mg/mL, aliquoted, and stored at 4°C protected from light.

### Plasmids for ratiometric phluorin-tagged proteins and cell transfection

Plasmids encoding for Golgi-localized proteins tagged with ratiometric pHluorin were provided by Terry E. Machen (University of California-Berkeley, Berkeley, CA) (Machen et al., 2003) and Yusuke Maeda (Osaka University, Suita, Osaka, Japan) (Maeda et al., 2008), the latter through an agreement with the Memorial Sloan Kettering Cancer Center (New York, NY). The plasmid encoding for transmembrane domains 1–3 of human Na^+^/H^+^ exchanger 6 (NHE6) fused to pHluorin2 (hNHE6-TM1–3-pHluorin2), results for which are shown herein, was generated using the pHluorin2 vector (Mahon, 2011) and primers listed in Table S1. The PCR products for NHE6 and pHluorin2 were digested using *Bam*HI restriction enzyme, ligated, and T-A cloned into pcDNA6.2-EmGFP/TOPO. The pcDNA6.2-EmGFP/TOPO vector was employed to take advantage of the CMV promotor; pHluorin2 has a stop codon, thus EmGFP will not be expressed.

With respect to transfection of plasmids, for our studies we used either HAP1 cells or mouse primary hippocampal neurons and the transfection reagent Lipofectamine® 2000 (ThermoFisher Scientific). The manufacturer's protocol was followed, using a plasmid DNA to reagent ratio of 1:2. However, a variety of transfection reagents and methods exist, any of which might be acceptable so long as the method provides for a reasonable transfection efficiency in the chosen cell type. Steps for a typical transfection include.

1. Passage cultured adherent cells the day before transfection; or plate primary cells a sufficient number of days in advance such that they are at the desired days *in vitro* (DIV) growth date at the time of transfection.
2. Plate cells on 35 mm glass bottom dishes so as to achieve a confluency (for adherent cultured cells) of 70–90% at the time of transfection. As an example, this is estimated at 3 × 10^5^ to 5 × 10^5^ for HAP1 cells and at 1.3 × 10^5^ for primary cultures of mouse hippocampal neurons.
3. Transfect cells using the desired transfection reagent and method.

### Fluorescent dye LysoSensor™

LysoSensor™ Yellow/Blue DND-160 was from ThermoFisher Scientific. A 1 mM stock solution was prepared in anhydrous dimethyl sulfoxide (DMSO), aliquoted, and stored in the freezer (−5 to −30°C) protected from light. For cases in which fluorescence measurements were made using a microplate reader, a SpectraMax® M5 Microplate Reader equipped with SoftMax® Pro V5 software (Molecular Devices) was used. The 96 well cell culture microplates were from Greiner Bio-One (Kremsmünster, Austria).

### Confocal microscopy

A Zeiss LSM 710 confocal laser scanning microscope and ZEN imaging software (ZEISS) were used for our studies. Additionally, during imaging, cells were maintained in a CO_2_ chamber held at 37°C^2^. Cells were first located using a 10X or 20X objective. Upon identifying an appropriate field of view, images were then acquired using a 63X oil objective. For fluorescence image acquisition, laser and filter settings were adjusted according to the fluorescence excitation and emission requirements of the experimental setup and reagents (Table 2). Separate tracks were set to avoid signal crossing and the tracks were set to switch every line. Digital images were acquired at a frame size of 1,024 × 1,024 pixels. The master gain was set such that pixels were at maximal saturation without being oversaturated.

**Table 2:** Peak excitation and emission wavelengths of reagents for measuring of intraorganellar pH.

**Reagent**	**Excitation peak (nm)**	**Emission peak (nm)**
Fluorescein isothiocyanate (FITC)	490	525
Alexa Fluor® 546	556	573
Ratiometric pHluorin	395 and 475	508
LysoSensor™ Yellow/Blue DND-160	329 and 384	440 and 540

### Flow cytometry/fluorescence-activated cell sorting (FACS)

For semi-adherent or non-adherent cells, flow cytometry can be used as an alternative to confocal microscopy for acquiring and analyzing data relating to ratiometric measurement of endocytosis of fluorescent conjugates of transferrin (Dunn and Maxfield, 2003). For our FACS-based analysis studies, we used the BD Influx™ cell sorter (BD Biosciences). We also used 5 mL polystyrene round-bottom tubes with cell-strainer caps and 5 mL polypropylene round-bottom tubes.

### Software for data analysis

Fluorescence intensities were quantified using the software programs ImageJ (NIH) for confocal microscopy images, FlowJo™ (Ashland, OR) for FACS data, and SoftMax® Pro V5 (Molecular Devices) for data based on reading of microplates. Data were exported to a Microsoft Excel spreadsheet. The pH-values of organelles were determined by fitting data to the pH calibration curves generated concurrently with each set of experiments. Data are presented as the average ± standard error of the mean (SEM).

## Stepwise procedures

### Ratiometric measurement of fluorescent conjugates of transferrin

This method takes advantage of a cell's endogenous endocytic pathway and the ubiquitous need for iron (Aisen and Listowsky, 1980; Sheftel et al., 2012). Endocytosis of transferrin, the iron-binding protein facilitating iron uptake in mammalian cells, and intracellular trafficking of the transferrin-transferrin receptor complex has long been studied. As such, the endocytic vesicle trafficking pathway of this ligand-receptor complex, namely, from early endosomes to recycling endosomes and back to the cell surface, is well known (Dautry-Varsat et al., 1983; Klausner et al., 1983; Maxfield and McGraw, 2004; Mayle et al., 2012). Furthermore, the pH of endocytic organelles involved in this trafficking has been determined for a variety of cell types, including through use of ratiometric fluorescence imaging or flow cytometry, with early studies performed in the 1980s and 1990s (Yamashiro et al., 1984; Sipe and Murphy, 1987; Sipe et al., 1991; Presley et al., 1993; Dunn et al., 1994).

In the protocols described below, cells are incubated simultaneously with two different fluorescent conjugates—one pH-sensitive and the other pH-insensitive—of transferrin. The fluorescently labeled transferrin binds to its cognate transferrin receptor at the cell's plasma membrane. This binding event signals the endocytosis of the transferrin-transferrin receptor complex, which then, depending on the time frame allowed for transferrin loading and the cell type, is trafficked to early endosomes and recycling endosomes (Mayle et al., 2012; Reineke et al., 2015). Experimentally, the amount of time allowed for incubation with the fluorescent conjugates of transferrin affects which endosomal compartment(s) will be labeled. Here, based on the indicated incubation times, two related protocols for measuring the pH of early endosomes are provided, one for cases in which confocal microscopy is used as a means for acquiring data (i.e., adherent cells) and one for cases in which flow cytometry/FACS might be more appropriate as a means for acquiring data (i.e., semi-adherent/non-adherent cells).

#### Confocal microscopy-based protocol

##### Generation of pH calibration curve

###### Transferrin loading

1. One day before the experiment, passage cells and seed cells into a 35 mm glass bottom dish at a density of ~3 × 10^5^ to ~5 × 10^5^. Alternatively, for primary cultures, plate cells a sufficient number of days in advance such that they are at the desired DIV growth date at the time of the experiment.
2. On the day of the experiment, incubate cells in warm serum-free cell culture medium for 30 min at 37°C to remove any residual transferrin.
3. Incubate cells for 30 min at 37°C in normal cell culture medium containing 66 μg/mL FITC-Tfn and 33 μg/mL Alexa Fluor® 546-Tfn (1 mL/dish). (See Table 3 for stock solutions and respective amounts for generating the indicated final concentrations of FITC-Tfn and Alexa Fluor® 546-Tfn in various volumes of cell culture medium.)
4. Wash cells four times with 1X phosphate-buffered saline (PBS) and leave cells in 1 mL of 1X PBS.

**Table 3:** Recipes for preparing FITC-Tfn and Alexa Fluor® 546-Tfn in cell culture medium.

	**5 mg/mL FITC-Tfn**	**5 mg/mL Alexa Fluor® 546-Tfn**	**Cell culture medium**
4.5 mL of medium	59.2 μL	30 μL	to 4.5 mL
6.0 mL of medium	79 μL	40 μL	to 6.0 mL
8.0 mL of medium	105.6 μL	52.8 μL	to 8.0 mL
Final concentration	66 μg/mL	33 μg/mL	–

##### Imaging

1. Set the confocal microscope at excitation and emission filter settings appropriate for imaging of FITC and Alexa Fluor® 546 fluorophores (Table 2). Set separate tracks to avoid signal crossing and set the tracks to switch every line during image acquisition.
2. Bring the dish of cells to the confocal microscope and find an appropriate region of cells for imaging using a low-power objective (e.g., 10X or 20X) and settings absent any activation of lasers (e.g., bright field)^2^.
3. Rinse cells once with the most alkaline of the pH calibration curve buffers (e.g., pH 7.5).
4. Incubate cells for 2 min in 1 mL of the first pH calibration curve buffer for imaging.
5. Collect four images of cells using a high-power objective (e.g., 63X) relatively quickly and taking care not to shift the horizontal plane so as to help ensure that images do not get out of register.^3^
6. Rinse cells once with 1X PBS and twice with the next pH calibration curve buffer in line, proceeding from most alkaline to most acidic.
7. Incubate cells for 2 min in 1 mL of the pH calibration curve buffer used in step 6 for rinsing but now for imaging.
8. Repeat steps 5–7 until images have been collected for cells incubated in at least five of the pH calibration curve buffers. Ensure to proceed from most alkaline (pH 7.5) to most acidic (pH 3.5).

##### Image analysis and plotting of data

1. For each pH calibration curve buffer, open the collected raw images (e.g., LSM, TIFF) in ImageJ.
2. Select regions of interest within cells that are reflective of transferrin-labeled endosomal compartments and record the endosomal fluorescence intensity values. Additionally, select regions of interest outside of cells (i.e., background) and record the background fluorescence intensity values for each channel. For endosomal compartments, selection of regions of interest should be based on the Alexa Fluor® 546 signal; however, fluorescence intensity values should be recorded for both the FITC signal and Alexa Fluor® 546 signal.
3. Export the fluorescence intensity measurement data to Microsoft Excel.
4. Subtract the background fluorescence intensity values for each channel from the corresponding fluorescence intensity values relating to transferrin-labeled endosomes.
5. Calculate the background-subtracted fluorescence intensity ratio of FITC signal:Alexa Fluor® 546 signal for each region of interest and for each pH calibration curve buffer.
6. Calculate and plot the average FITC signal:Alexa Fluor® 546 signal ratio for each pH calibration curve buffer.
7. Fit the data so as to generate a pH calibration curve for use in determining pH-values based on experimental data^1^.

##### Measurement of endosomal pH

###### Transferrin loading

1. One day before the experiment, passage cells and seed cells into a 35 mm glass bottom dish at a density of ~3 × 10^5^ to ~5 × 10^5^. Alternatively, for primary cultures, plate cells a sufficient number of days in advance such that they are at the desired DIV growth date at the time of the experiment.
2. On the day of the experiment, incubate cells in warm serum-free cell culture medium for 30 min at 37°C to remove any residual transferrin.
3. Incubate cells for 10 min at 37°C in normal cell culture medium containing 66 μg/mL FITC-Tfn and 33 μg/mL Alexa Fluor® 546-Tfn (1 mL/dish) (Table 3)^4^.
4. Wash cells four times with 1X PBS.
5. Incubate cells in 1 mL of phenol red-free cell culture medium. (Use of such cell culture medium is to reduce autofluorescence.)

##### Imaging

1. Set the confocal microscope settings to those used in collecting images to generate the pH calibration curve.
2. Bring the dish of cells to the confocal microscope and find an appropriate region of cells for imaging^2^.
3. Collect six to eight images of cells using a high-power objective (e.g., 63X) and the same settings as those used in collecting images to generate the pH calibration curve. Images should be acquired over a time span of not more than 7–8 min and taking care not to shift the horizontal plane so as to help ensure that images do not get out of register^3, 4^.

##### Image analysis and fitting of data

1. Open the collected raw images (e.g., LSM, TIFF) in ImageJ.
2. Select regions of interest within cells that are reflective of transferrin-labeled endosomal compartments and record the endosomal fluorescence intensity values. Additionally, select regions of interest outside of cells (i.e., background) and record the background fluorescence intensity values for each channel. For endosomal compartments, selection of regions of interest should be based on the Alexa Fluor® 546 signal; however, fluorescence intensity values should be recorded for both the FITC signal and Alexa Fluor® 546 signal.
3. Export the fluorescence intensity measurement data to Microsoft Excel.
4. Subtract the background fluorescence intensity values for each channel from the corresponding fluorescence intensity values relating to transferrin-labeled endosomes.
5. Calculate the background-subtracted fluorescence intensity ratio of FITC signal:Alexa Fluor® 546 signal for each region of interest (i.e., endosomes).
6. Calculate the pH of each region of interest using the pH calibration curve generated in parallel with the experiment.
7. Calculate the average pH for all measured regions of interest across all cells, thereby resulting in an average endosomal pH.

#### Flow cytometry-based/FACS-based protocol

##### Generation of pH calibration curve

###### Transferrin loading

1. One day before the experiment, passage cells and seed cells into a six well plate at a density of ~3 × 10^5^ to ~5 × 10^5^. Alternatively, for primary cultures, plate cells a sufficient number of days in advance such that they are at the desired DIV growth date at the time of the experiment. One well of cells is to be left untreated as a negative control (i.e., background) and for setting of flow cytometer settings; the other five wells of cells are treated as outlined below.
2. On the day of the experiment, incubate cells in warm serum-free cell culture medium for 30 min at 37°C to remove any residual transferrin.
3. Incubate cells for 30 min at 37°C in normal cell culture medium containing 66 μg/mL FITC-Tfn and 33 μg/mL Alexa Fluor® 546-Tfn (1 mL/well) (Table 3).
4. Wash cells in all wells twice with cold 1X PBS.
5. Trypsinize cells in all wells and transfer cells to six Eppendorf tubes for flow cytometry/FACS.
6. Wash cells four times with 1X PBS. Centrifuge cells at 300–400 × *g* for 1 min between washes to gently pellet cells.
7. For untreated cells, (a) discard the final supernatant from step 6, (b) resuspend cells in 400 μL of phenol red-free cell culture medium, (c) process cells through a cell strainer to generate single-cell populations, and (d) place cells on ice until used in preparing the flow cytometer for FACS-based analysis.
8. For treated cells, discard the final supernatant from step 6 just prior to step 2 below.

##### Flow cytometry/FACS

1. Using the tube of untreated cells, prepare the flow cytometer for FACS-based analysis using excitation and emission filter settings appropriate for sorting on FITC and Alexa Fluor® 546 fluorophores (Table 2).
2. For each of the five tubes of treated cells, rinse cells twice with one of the pH calibration curve buffers, selecting a buffer of a different pH for each of the five tubes (e.g., pH 7.0, 6.5, 6.0, 5.5, and 5.0). Centrifuge cells at 300–400 × *g* for 1 min between washes to gently pellet cells.
3. Discard the final supernatant from step 2 and resuspend cells in 400 μL of the pH calibration curve buffer used for rinsing. Quickly proceed to the next step.
4. Process cells through a cell strainer to generate single-cell populations just prior to their use for FACS-based analysis.
5. Rapidly analyze the cells by FACS using settings determined in step 1 for sorting of cells that have endocytosed both FITC-Tfn and Alexa Fluor® 546-Tfn.

##### Cell analysis and plotting of data

1. For each pH calibration curve buffer, analyze the sorted cells using appropriate software (e.g., FlowJo™). Ensure that the sample of untreated cells is also analyzed, namely, as a negative control for obtaining background fluorescence intensity data.
2. Export the mean fluorescence intensity data for each the FITC signal and the Alexa Fluor® 546 signal for each pH calibration curve buffer to Microsoft Excel.
3. Subtract the background fluorescence intensity values for each channel (i.e., untreated cells) from the corresponding fluorescence intensity values relating to transferrin-labeled endosomes (i.e., treated cells).
4. Calculate the ratio of background-subtracted mean fluorescence intensity for the FITC signal vs. the Alexa Fluor® 546 signal for each pH calibration curve buffer.
5. Calculate and plot the average FITC signal:Alexa Fluor® 546 signal ratio for each pH calibration curve buffer.
6. Fit the data so as to generate a pH calibration curve for use in determining pH-values based on experimental data^1^.

##### Measurement of endosomal pH

###### Transferrin loading

1. One day before the experiment, passage cells and seed cells into a six well plate at a density of ~3 × 10^5^ to ~5 × 10^5^. Alternatively, for primary cultures, plate cells a sufficient number of days in advance such that they are at the desired DIV growth date at the time of the experiment.
2. On the day of the experiment, incubate cells in warm serum-free cell culture medium for 30 min at 37°C to remove any residual transferrin.
3. Incubate cells for 10 min at 37°C in normal cell culture medium containing 66 μg/mL FITC-Tfn and 33 μg/mL Alexa Fluor® 546-Tfn (1 mL/well) (Table 3)^4^.
4. Wash cells twice with cold 1X PBS.
5. Trypsinize cells in all wells and transfer cells to six Eppendorf tubes for flow cytometry/FACS.
6. Wash cells four times with 1X PBS. Centrifuge cells at 300–400 × *g* for 1 min between washes to gently pellet cells. Discard the final supernatant just prior to step 2 below.

##### Flow cytometry/FACS

1. Prepare the flow cytometer for FACS-based analysis using the same settings as those used for sorting of cells in generating the pH calibration curve.
2. Resuspend cells in 400 μL of phenol red-free cell culture medium.
3. Process cells through a cell strainer to generate single-cell populations just prior to their use for FACS-based analysis.
4. Rapidly analyze the cells by FACS using the same settings as those used for sorting of cells in generating the pH calibration curve.

##### Cell analysis and fitting of data

1. Analyze the sorted cells using appropriate software (e.g., FlowJo™). Ensure that the sample of untreated cells is also analyzed, namely, as a negative control for obtaining background fluorescence intensity data.
2. Export the mean fluorescence intensity data for each the FITC signal and the Alexa Fluor® 546 signal for each population of sorted cells to Microsoft Excel.
3. Subtract the background fluorescence intensity values for each channel (i.e., untreated cells) from the corresponding fluorescence intensity values relating to transferrin-labeled endosomes (i.e., treated cells).
4. Calculate the ratio of background-subtracted mean fluorescence intensity for the FITC signal vs. the Alexa Fluor® 546 signal for each population of cells.
5. Calculate the pH of organelles labeled within the sorted cells using the pH calibration curve generated in parallel with the experiment.
6. Calculate the average pH of organelles labeled within the sorted cells based on data for all analyzed replicates, thereby resulting in an average endosomal pH.

### Measurement of intrinsically fluorescent ratiometric phluorin fusion proteins

Researchers have taken advantage of the pH-dependent nature of green fluorescent protein (GFP) to detect intracellular and intraorganellar acidity (Grubb and Burrone, 2009; Bencina, 2013; Grillo-Hill et al., 2014). Depending on the protonation state of the chromophore, wild-type GFP exists in either of two alternative conformations and therefore has a bimodal excitation spectrum with peaks at 395 nm (protonated) and 475 nm (deprotonated) (Chattoraj et al., 1996; Brejc et al., 1997; Palm et al., 1997). pH-dependent switching between the states can be enhanced by introducing specific amino-acid substitutions, thereby allowing for the development of useful genetically encoded, fluorescent protein-based biosensors for detecting changes in pH within cells (Kneen et al., 1998; Miesenbock et al., 1998). Some of such GFP derivatives have been termed “pHluorins,” with classes of pHluorins including ecliptic pHluorin (i.e., pH-dependent change in the intensity of emission at a single excitation wavelength) and ratiometric pHluorin (i.e., pH-dependent change in the ratio of the intensity of emission at a shorter excitation wavelength vs. the intensity of emission at a longer excitation wavelength) (Miesenbock et al., 1998; Bencina, 2013). Here, we provide a protocol for measurement of intraorganellar pH based on use of ratiometric pHluorin-tagged proteins localized to the luminal domain of specific intracellular compartments.

#### Generation of pH calibration curve

##### Transfection

1. Plate cells on 35 mm glass bottom dishes so as to achieve a confluency (for adherent cultured cells) of 70–90% at the time of transfection. Alternatively, for primary cultures, plate cells a sufficient number of days in advance such that they are at the desired DIV growth date at the time of the experiment.
2. Transfect cells with a plasmid encoding for a ratiometric pHluorin-tagged protein of interest following standard procedures that will allow for a reasonable transfection efficiency for the given cell type.
3. Incubate cells for at least 20 to 24 h to allow for protein expression.
4. Rinse cells once with 1X PBS and leave cells in 1 mL of 1X PBS.

##### Imaging

1. Set the confocal microscope at excitation and emission filter settings appropriate for imaging of ratiometric pHluorin (Table 2). Set separate tracks to avoid signal crossing and set the tracks to switch every line during image acquisition.
2. Bring the dish of cells to the confocal microscope and find an appropriate region of ratiometric pHluorin-expressing cells for imaging using a low-power objective (e.g., 10X or 20X)^2^.
3. Rinse cells once with the most alkaline of the pH calibration curve buffers (e.g., pH 7.5).
4. Incubate cells for 2 min in 1 mL of the first pH calibration curve buffer for imaging.
5. Collect images of at least 10 cells using a high-power objective (e.g., 63X) relatively quickly and taking care not to shift the horizontal plane so as to help ensure that images do not get out of register^3^.
6. Rinse cells once with 1X PBS and twice with the next pH calibration curve buffer in line, proceeding from most alkaline to most acidic.
7. Incubate cells for 2 min in 1 mL of the pH calibration curve buffer used in step 6 for rinsing but now for imaging.
8. Repeat steps 5–7 until images have been collected for cells incubated in at least five of the pH calibration curve buffers. Ensure to proceed from most alkaline (pH 7.5) to most acidic (pH 3.5).

##### Image analysis and plotting of data

1. For each pH calibration curve buffer, open the collected raw images (e.g., LSM, TIFF) in ImageJ.
2. Select regions of interest within cells that are reflective of ratiometric pHluorin-labeled organelles of interest and record the fluorescence intensity values of such regions. Fluorescence intensity values should be recorded for both emission at a shorter excitation wavelength (e.g., 410 nm) and emission at a longer excitation wavelength (e.g., 470 nm). Additionally, select regions of interest outside of cells (i.e., background) and record the background fluorescence intensity values for each excitation wavelength.
3. Export the fluorescence intensity measurement data to Microsoft Excel.
4. Subtract the background fluorescence intensity values for each excitation wavelength from the corresponding fluorescence intensity values relating to ratiometric pHluorin-labeled organelles.
5. Calculate the background-subtracted fluorescence intensity ratio of intensity of emission at shorter excitation wavelength (e.g., 410 nm):intensity of emission at longer excitation wavelength (e.g., 410 nm) for each region of interest and for each pH calibration curve buffer.
6. Calculate and plot the average fluorescence intensity ratio for each pH calibration curve buffer.
7. Fit the data so as to generate a pH calibration curve for use in determining pH-values based on experimental data^1^.

#### Measurement of intraorganellar pH

##### Transfection

1. Plate cells on 35 mm glass bottom dishes so as to achieve a confluency (for adherent cultured cells) of 70–90% at the time of transfection. Alternatively, for primary cultures, plate cells a sufficient number of days in advance such that they are at the desired DIV growth date at the time of the experiment.
2. Transfect cells with a plasmid encoding for a ratiometric pHluorin-tagged protein of interest following standard procedures that will allow for a reasonable transfection efficiency for the given cell type.
3. Incubate cells for at least 20 to 24 h to allow for protein expression.
4. Rinse cells once with 1X PBS.
5. Incubate cells in 1 mL of phenol red-free cell culture medium. (Use of such cell culture medium is to reduce autofluorescence.)

##### Imaging

1. Set the confocal microscope settings to those used in collecting images to generate the pH calibration curve.
2. Bring the dish of cells to the confocal microscope and find an appropriate region of ratiometric pHluorin-expressing cells for imaging^2^.
3. Collect images of 20–40 cells using a high-power objective (e.g., 63X) and the same settings as those used in collecting images to generate the pH calibration curve. Images should be acquired relatively quickly and taking care not to shift the horizontal plane so as to help ensure that images do not get out of register^3^.

##### Image analysis and fitting of data

1. Open the collected raw images (e.g., LSM, TIFF) in ImageJ.
2. Select regions of interest within cells that are reflective of ratiometric pHluorin-labeled organelles of interest and record the fluorescence intensity values of such regions. Fluorescence intensity values should be recorded for both emission at a shorter excitation wavelength (e.g., 410 nm) and emission at a longer excitation wavelength (e.g., 470 nm). Additionally, select regions of interest outside of cells (i.e., background) and record the background fluorescence intensity values for each excitation wavelength.
3. Export the fluorescence intensity measurement data to Microsoft Excel.
4. Subtract the background fluorescence intensity values for each excitation wavelength from the corresponding fluorescence intensity values relating to ratiometric pHluorin-labeled organelles.
5. Calculate the background-subtracted fluorescence intensity ratio of intensity of emission at shorter excitation wavelength (e.g., 410 nm):intensity of emission at longer excitation wavelength (e.g., 470 nm) for each region of interest.
6. Calculate the pH of each region of interest using the pH calibration curve generated in parallel with the experiment.
7. Calculate the average pH for all measured regions of interest across all cells, thereby resulting in an average intraorganellar pH reflective of the organelle targeted by the ratiometric pHluorin fusion protein.

### Measurement of the fluorescent dye LysoSensor™

A characteristic feature of lysosomes, and one with great relevance to their function, is a highly acidic luminal pH (pH ~4.5–~5.5) (Luzio et al., 2007; Casey et al., 2010). The LysoSensor™ family of fluorescent dyes (ThermoFisher Scientific) provides a means for fluorescence-based measurement of lysosomal pH. These dyes are membrane-permeant weak bases that accumulate in the lumen of acidic organelles upon protonation. Additionally, the protonation relieves the inherent fluorescence quenching of the dye, which subsequently results in an increase in fluorescence intensity. As weak bases, a note of caution with their use is, however, the potential for an alkalinizing effect on intraorganellar pH (Life Technologies, 2013; Guha et al., 2014). Here, we provide protocols for using the LysoSensor™ family member LysoSensor™ Yellow/Blue DND-160. This family member in particular allows for ratiometric measurement of intraorganellar pH through use of dual-wavelength fluorescence-based analysis. In living cells, the fluorescent dye produces yellow fluorescence in acidic environments, such as lysosomes, whereas it produces blue fluorescence in neutral environments. Two related protocols are provided, one for cases in which confocal microscopy is used as a means for acquiring data and one for cases in which data are acquired using a microplate reader.

#### Confocal microscopy-based method

##### Generation of pH calibration curve

###### Loading of LysoSensor™ dye

1. One day before the experiment, passage cells and seed cells into a 35 mm glass bottom dish at a density of ~3 × 10^5^ to ~5 × 10^5^. Alternatively, for primary cultures, plate cells a sufficient number of days in advance such that they are at the desired DIV growth date at the time of the experiment.
2. On the day of the experiment, dilute the LysoSensor™ Yellow/Blue DND-160 stock solution (1 mM) to the final working concentration in normal cell culture medium. A recommended working concentration is at least 1 μM, but may be from 2 to 5 μM.
3. Incubate cells at 37°C in 1 mL of pre-warmed, normal cell culture medium containing LysoSensor™ Yellow/Blue DND-160 diluted to the working concentration (step 2). A suggested time period for incubation is 1–5 min^5^.
4. Rinse cells twice with 1X PBS and leave cells in 1 mL of 1X PBS.

##### Imaging

1. Set the confocal microscope at excitation and emission filter settings appropriate for imaging of LysoSensor™ Yellow/Blue DND-160 (Table 2). Set separate tracks to avoid signal crossing and set the tracks to switch every line during image acquisition.
2. Bring the dish of cells to the confocal microscope and find an appropriate region of cells for imaging using a low-power objective (e.g., 10X or 20X)^2^.
3. Rinse cells once with the most alkaline of the pH calibration curve buffers (e.g., pH 7.5).
4. Incubate cells for 2 min in 1 mL of the first pH calibration curve buffer for imaging.
5. Collect four images of cells using a high-power objective (e.g., 63X) relatively quickly and taking care not to shift the horizontal plane so as to help ensure that images do not get out of register^3^.
6. Rinse cells once with 1X PBS and twice with the next pH calibration curve buffer in line, proceeding from most alkaline to most acidic.
7. Incubate cells for 2 min in 1 mL of the pH calibration curve buffer used in step 6 for rinsing but now for imaging.
8. Repeat steps 5–7 until images have been collected for cells incubated in at least five of the pH calibration curve buffers. Ensure to proceed from most alkaline (pH 7.5) to most acidic (pH 3.5).

##### Image analysis and plotting of data

1. For each pH calibration curve buffer, open the collected raw images (e.g., LSM, TIFF) in ImageJ.
2. Select regions of interest within cells that are reflective of LysoSensor™ Yellow/Blue DND-160-labeled organelles of interest and record the fluorescence intensity values of such regions. Fluorescence intensity values should be recorded for emissions at both wavelengths (e.g., 440 and 540 nm). Additionally, select regions of interest outside of cells (i.e., background) and record the background fluorescence intensity values for each emission wavelength.
3. Export the fluorescence intensity measurement data to Microsoft Excel.
4. Subtract the background fluorescence intensity values for each emission wavelength from the corresponding fluorescence intensity values relating to LysoSensor™ Yellow/Blue DND-160-labeled organelles.
5. Calculate the background-subtracted fluorescence intensity ratio of intensity of emission at shorter wavelength (e.g., 440 nm):intensity of emission at longer wavelength (e.g., 540 nm) for each region of interest and for each pH calibration curve buffer.
6. Calculate and plot the average fluorescence intensity ratio for each pH calibration curve buffer.
7. Fit the data so as to generate a pH calibration curve for use in determining pH-values based on experimental data^1^.

##### Measurement of intraorganellar pH

###### Loading of LysoSensor™ dye

1. One day before the experiment, passage cells and seed cells into a 35 mm glass bottom dish at a density of ~3 × 10^5^ to ~5 × 10^5^. Alternatively, for primary cultures, plate cells a sufficient number of days in advance such that they are at the desired DIV growth date at the time of the experiment.
2. On the day of the experiment, dilute the LysoSensor™ Yellow/Blue DND-160 stock solution (1 mM) to the final working concentration in normal cell culture medium.
3. Incubate cells for the same time period as used in generating the pH calibration curve (e.g., 1–5 min) at 37°C in 1 mL of pre-warmed, normal cell culture medium containing LysoSensor™ Yellow/Blue DND-160 diluted to the working concentration (e.g., 1 μM) (step 2).
4. Rinse cells twice with 1X PBS.
5. Incubate cells in 1 mL of phenol red-free cell culture medium. (Use of such cell culture medium is to reduce autofluorescence.)

##### Imaging

1. Set the confocal microscope settings to those used in collecting images to generate the pH calibration curve.
2. Bring the dish of cells to the confocal microscope and find an appropriate region of cells for imaging^2^.
3. Collect images of at least 10 cells using a high-power objective (e.g., 63X) and the same settings as those used in collecting images to generate the pH calibration curve. Images should be acquired over a time span of not more than 10 min and taking care not to shift the horizontal plane so as to help ensure that images do not get out of register^3^.

##### Image analysis and fitting of data

1. Open the collected raw images (e.g., LSM, TIFF) in ImageJ.
2. Select regions of interest within cells that are reflective of LysoSensor™ Yellow/Blue DND-160-labeled organelles of interest and record the fluorescence intensity values of such regions. Fluorescence intensity values should be recorded for emissions at both wavelengths (e.g., 440 nm and 540 nm). Additionally, select regions of interest outside of cells (i.e., background) and record the background fluorescence intensity values for each emission wavelength.
3. Export the fluorescence intensity measurement data to Microsoft Excel.
4. Subtract the background fluorescence intensity values for each emission wavelength from the corresponding fluorescence intensity values relating to LysoSensor™ Yellow/Blue DND-160-labeled organelles.
5. Calculate the background-subtracted fluorescence intensity ratio of intensity of emission at shorter wavelength (e.g., 440 nm):intensity of emission at longer wavelength (e.g., 540 nm) for each region of interest.
6. Calculate the pH of each region of interest using the pH calibration curve generated in parallel with the experiment.
7. Calculate the average pH for all measured regions of interest across all cells, thereby resulting in an average intraorganellar pH reflective of the selected LysoSensor™ Yellow/Blue DND-160-labeled organelles.

#### Microplate reader-based method

##### Generation of pH calibration curve

###### Loading of LysoSensor™ dye

1. One day before the experiment, passage cells and seed cells into a 96 well plate at a density of ~3 × 10^4^. Alternatively, for primary cultures, plate cells a sufficient number of days in advance such that they are at the desired DIV growth date at the time of the experiment. Note, both generation of the pH calibration curve and collection of experimental data can be performed simultaneously in a single 96 well plate.
2. On the day of the experiment, dilute the LysoSensor™ Yellow/Blue DND-160 stock solution (1 mM) to the final working concentration in normal cell culture medium. A recommended working concentration is at least 1 μM, but may be from 2 to 5 μM.
3. Incubate cells at 37°C in 1 mL of pre-warmed, normal cell culture medium containing LysoSensor™ Yellow/Blue DND-160 diluted to the working concentration (step 2). A suggested time period for incubation is 1–5 min^5^.
4. Rinse cells twice with 1X PBS and leave cells in 100 μL of 1X PBS.

##### Reading of microplate

1. Set the microplate reader at excitation and emission settings appropriate for reading of LysoSensor™ Yellow/Blue DND-160 fluorescence (Table 2).
2. Using multiple wells of cells for each pH calibration curve buffer (e.g., three wells per buffer), rinse each well of cells once with its respective pH calibration curve buffer.
3. Incubate each well of cells for 10 min in 100 μL of its respective pH calibration curve buffer for reading. Allocate wells of cells so as to ensure that, within the single plate, incubation of cells in at least five of the pH calibration curve buffers has been accounted for.
4. Collect readouts of cell fluorescence relatively quickly in triplicate.

##### Microplate reading results analysis and plotting of data

1. For each pH calibration curve buffer, export the fluorescence intensity measurement data based on the microplate readings and for emissions at both wavelengths (e.g., 440 and 540 nm) to Microsoft Excel.
2. Calculate the fluorescence intensity ratio of intensity of emission at shorter wavelength (e.g., 440 nm):intensity of emission at longer wavelength (e.g., 540 nm) for each pH calibration curve buffer.
3. Calculate and plot the average fluorescence intensity ratio for each pH calibration curve buffer.
4. Fit the data so as to generate a pH calibration curve for use in determining pH-values based on experimental data^1^.

##### Measurement of intraorganellar pH

###### Loading of LysoSensor™ dye

1. One day before the experiment, passage cells and seed cells into a 96 well plate at a density of ~3 × 10^4^. Alternatively, for primary cultures, plate cells a sufficient number of days in advance such that they are at the desired DIV growth date at the time of the experiment. Note, both generation of the pH calibration curve and collection of experimental data can be performed simultaneously in a single 96 well plate.
2. On the day of the experiment, dilute the LysoSensor™ Yellow/Blue DND-160 stock solution (1 mM) to the final working concentration in normal cell culture medium.
3. Incubate cells for the same time period as used in generating the pH calibration curve (e.g., 1–5 min) at 37°C in 100 μL of pre-warmed, normal cell culture medium containing LysoSensor™ Yellow/Blue DND-160 diluted to the working concentration (e.g., 1 μM) (step 2).
4. Rinse cells twice with 1X PBS.
5. Incubate cells in 100 μL of phenol red-free cell culture medium. (Use of such cell culture medium is to reduce autofluorescence.)

##### Reading of microplate

1. Set the microplate reader settings to those used in generating the pH calibration curve.
2. Collect readouts of cell fluorescence relatively quickly in triplicate.

##### Microplate reading results analysis and fitting of data

1. Export the fluorescence intensity measurement data based on the microplate readings and for emissions at both wavelengths (e.g., 440 and 540 nm) to Microsoft Excel.
2. Calculate the fluorescence intensity ratio of intensity of emission at shorter wavelength (e.g., 440 nm):intensity of emission at longer wavelength (e.g., 540 nm).
3. Calculate the pH of cells using the pH calibration curve generated in parallel with the experiment.
4. Calculate the average pH for all readouts, thereby resulting in an average whole-cell intraorganellar pH reflective of all LysoSensor™ Yellow/Blue DND-160-labeled organelles combined.

## Anticipated results

### Ratiometric measurement of fluorescent conjugates of transferrin

Transferrin transports iron from sites of absorption and storage to tissue cells by way of blood plasma (Aisen and Listowsky, 1980; Sheftel et al., 2012). Delivery of iron into cells involves binding of ferro-transferrin (or holo-transferrin) to its cell surface receptor, namely, the transferrin receptor, and subsequent internalization of the ligand–receptor complex through receptor-mediated endocytosis. At the acidic pH of endosomes, iron is released from transferrin for delivery to the cytosol whereas the transferrin-transferrin receptor complex remains associated for recycling back to the cell surface (Dautry-Varsat et al., 1983; Klausner et al., 1983; Maxfield and McGraw, 2004; Mayle et al., 2012). Since transferrin remains associated with its receptor as it recycles, fluorescent conjugates of transferrin can be used to monitor the transferrin–transferrin receptor complex as it traverses the early endocytic and recycling pathways.

When pH-sensitive and pH-insensitive fluorescent conjugates are used simultaneously, either on the same transferrin molecule or on separate transferrin molecules, the pH of endosomal compartments can be measured ratiometrically. Indeed, a number of studies have been published using this method, in the 1980s and 1990s (Yamashiro et al., 1984; Sipe and Murphy, 1987; Sipe et al., 1991; Presley et al., 1993; Dunn et al., 1994) and more recently (Diering et al., 2013; Ouyang et al., 2013; Kondapalli et al., 2015; Prasad and Rao, 2015; Fan et al., 2016). A limitation of this method is, however, the potential and perhaps likelihood that multiple types of endosomal compartments (e.g., early endosomes, recycling endosomes) will be labeled at a given time point of analysis depending on the time allowed for incubation with the fluorescent conjugates of transferrin and organelle dynamics (Mayle et al., 2012; Reineke et al., 2015). Also, if the two different fluorophores, one pH-sensitive and the other pH-insensitive, are conjugated to two separate transferrin molecules, the potential exists for the two fluorescent conjugates of transferrin to exhibit differences in endocytic uptake and/or sorting and vesicle trafficking, which would then affect the fluorescence intensity ratios at different endosomal compartments.

**Figure 1:**
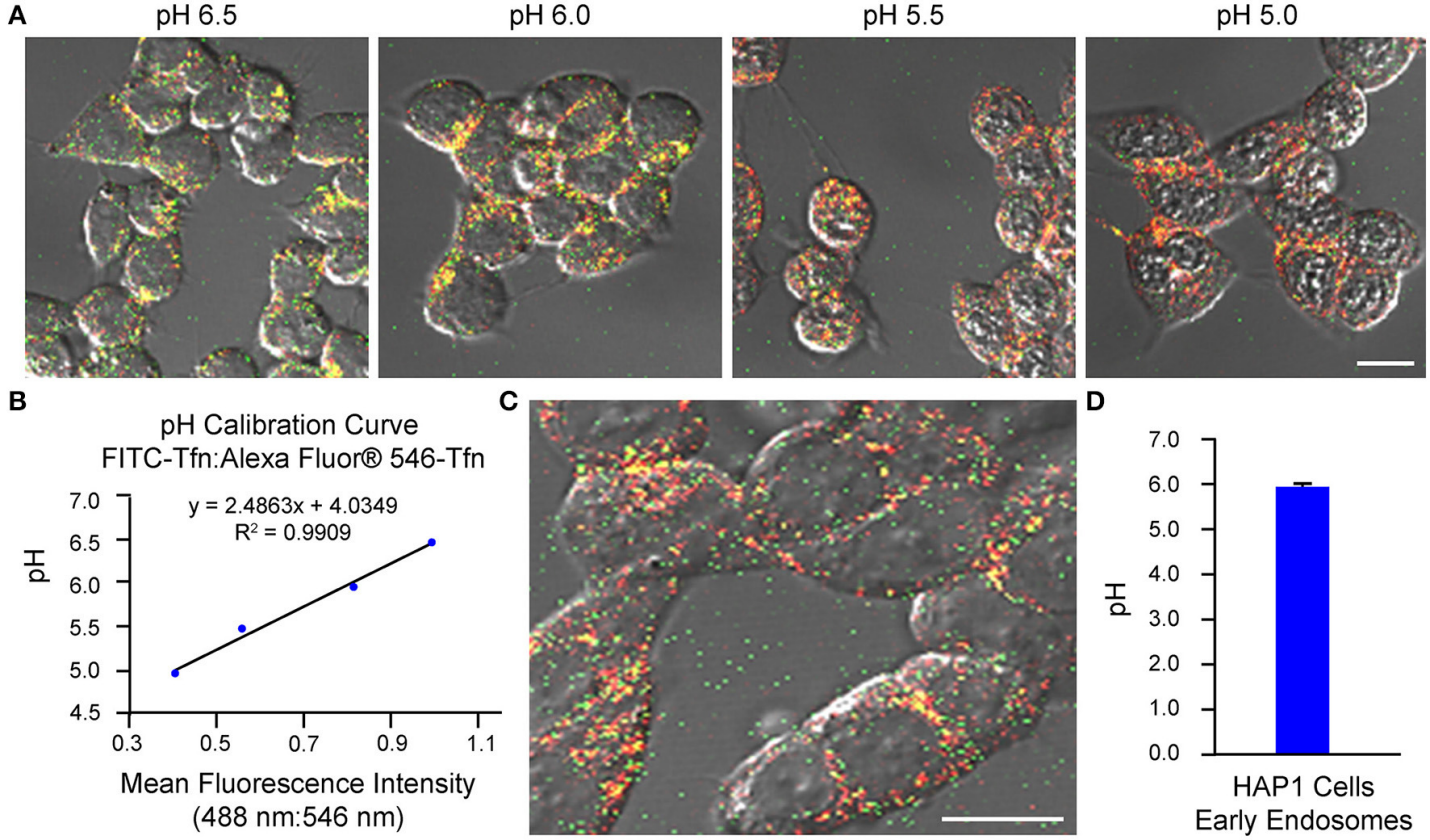
Use of confocal microscopy-based ratiometric measurement of fluorescent conjugates of transferrin to determine the pH of early endosomes in HAP1 cells. HAP1 cells were treated as described in the stepwise procedures, incubating cells with FITC-Tfn (66 μg/mL) and Alexa Fluor® 546-Tfn (33 μg/mL) for 30 min in generating the pH calibration curve and for 10 min in acquiring experimental data. Cells were subsequently analyzed using confocal microscopy and ImageJ software to determine fluorescence intensity ratios. Emissions were collected at 493–556 nm (FITC-Tfn) and 566–680 nm (Alexa Fluor® 546-Tfn) for excitations at 488 and 546 nm, respectively. **(A)** Fluorescence microscopy images of transferrin-treated HAP1 cells incubated in pH calibration curve buffers of the indicated pH-values. Scale bar, 10 μm. **(B)** Graph of the pH calibration curve generated based on fluorescence intensity measurements of regions of interest identified in HAP1 cells such as those shown in **(A)**. The pH calibration curve was generated in parallel with acquiring experimental data. **(C)** Representative fluorescence microscopy image of transferrin-treated HAP1 cells used for collecting experimental data. Scale bar, 10 μm. **(D)** Graph of the average pH of early endosomes in HAP1 cells, as determined by using confocal microscopy-based ratiometric measurement of fluorescent conjugates of transferrin. Fluorescence intensity ratios were converted to pH-values by fitting of data to the pH calibration curve shown in **(B)**. The average pH of early endosomes in HAP1 cells was calculated at 6.0 ± 0.1. Data are presented as the average ± SEM.

As an example of implementation of the above protocol for ratiometric measurement of pH-sensitive (FITC-Tfn) and pH-insensitive (Alexa Fluor® 546-Tfn) fluorescent conjugates of transferrin using confocal microscopy, we have used cells of the cultured cell line HAP1. We have also published on application of this method to primary cultures of mouse hippocampal neurons (Ouyang et al., 2013). It is noted, for experiments in which cells are analyzed using confocal microscopy, the two fluorophores (even if on the same molecule) may exhibit differences in susceptibility to and rates of photobleaching, which would then affect the fluorescence intensity ratios. To this end, the laser intensity and duration of imaging should be kept to a minimum, as should repeated excitations, while at the same time allowing for images of an adequate signal-to-noise ratio to be acquired. Results from implementation of the confocal microscopy-based method of analysis described herein are shown in Figure 1, with generation of the pH calibration curve shown in Figures 1A,B and measurement of endosomal pH shown in Figures 1C,D. Based on this example, the average pH of early endosomes in HAP1 cells was calculated at 6.0 ± 0.1.

**Figure 2:**
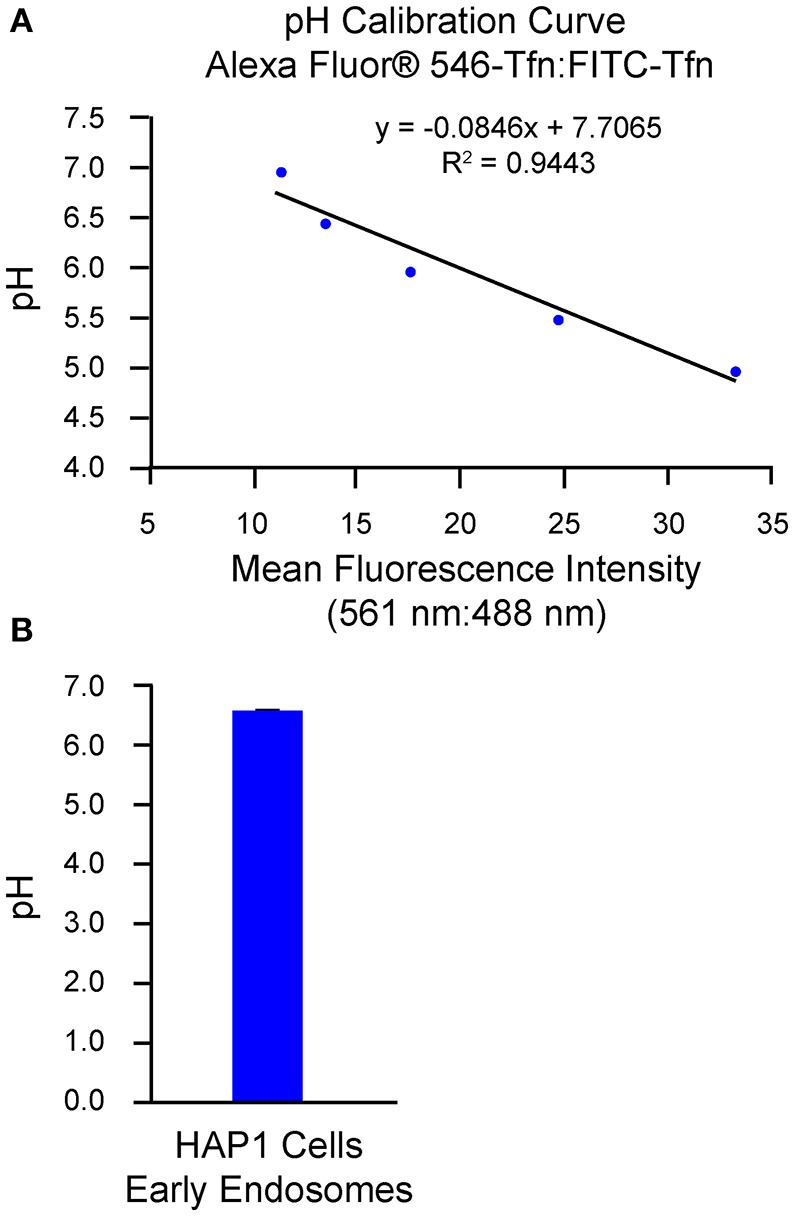
Use of flow cytometry-based/FACS-based ratiometric measurement of fluorescent conjugates of transferrin to determine the pH of early endosomes in HAP1 cells. HAP1 cells were treated as described in the stepwise procedures, incubating cells with FITC-Tfn (66 μg/mL) and Alexa Fluor® 546-Tfn (33 μg/mL) for 30 min in generating the pH calibration curve and for 10 min in acquiring experimental data. Cells were subsequently analyzed using flow cytometry/FACS and FlowJo™ software to determine fluorescence intensity ratios. Emissions were collected at 528–538 nm (FITC-Tfn) and 593–640 nm (Alexa Fluor® 546-Tfn) for excitations at 488 and 561 nm, respectively. **(A)** Graph of the pH calibration curve generated based on fluorescence intensity measurements of HAP1 cells incubated with pH calibration curve buffers of the indicated pH-values and analyzed by flow cytometry/FACS. The pH calibration curve was generated in parallel with acquiring experimental data. **(B)** Graph of the average pH of early endosomes in HAP1 cells, as determined by using flow cytometry-based/FACS-based ratiometric measurement of fluorescent conjugates of transferrin. Four replicates were performed, with each replicate containing 20,000 events. Fluorescence intensity ratios were converted to pH-values by fitting of data to the pH calibration curve shown in **(A)**. The average pH of early endosomes in HAP1 cells was calculated at 6.57 ± 0.01. Data are presented as the average ± SEM.

An alternative method of analysis regarding ratiometric measurement of fluorescent conjugates of transferrin is flow cytometry/FACS (Sipe and Murphy, 1987; Kondapalli et al., 2015; Prasad and Rao, 2015). Use of this method is of particular benefit when a need exists to analyze cells that are of a semi-adherent or non-adherent nature (Dunn and Maxfield, 2003). Additionally, many cells (i.e., thousands) can be measured for each histogram in a single time course and with limited photobleaching or photolysis, which can be a concern when imaging cells for extended periods. However, in contrast to the use of confocal microscopy as a means for analysis, discrete regions of interest cannot be selected when using flow cytometry/FACS and little to no spatial or morphological information is obtained. Also, fluorescence from non-specific extracellular ligand remaining bound after washes may contribute to the overall signal if not accounted for and excluded. To this end, one method to estimate the amount of fluorescence signal derived from surface-bound transferrin is through external application of a fluorophore-conjugated antibody against transferrin at specific time points of analysis and calculation of the percent transferrin remaining at the surface, as described in Sipe and Murphy (1987). Another approach is to remove transferrin bound at the surface through inclusion of an acid strip step in which, after incubation of cells with fluorophore-conjugated transferrin, cells are rinsed in PBS at pH 5.0 followed by a rinse in PBS at pH 7.0 (Kondapalli et al., 2013, 2015). Results from implementation of the flow cytometry-based/FACS-based method of analysis described herein are shown in Figure 2, with generation of the pH calibration curve shown in Figure 2A and measurement of endosomal pH shown in Figure 2B. Based on this example, the average pH of early endosomes in HAP1 cells was calculated at 6.57 ± 0.01.

### Measurement of intrinsically fluorescent ratiometric phluorin fusion proteins

The use of ratiometric pHluorin provides a genetically encoded means of monitoring intraorganellar pH. As such, the need for pre-loading of cells with a fluorescent probe is bypassed and the method is relatively non-invasive. Furthermore, because the pHluorin protein can be fused to the luminal domain of a protein of choice, this method allows for flexibility and specificity in the targeting of organelles for which one wishes to monitor intraorganellar pH. Transfection efficiency and protein expression levels and localization patterns can, however, present concerns and problems requiring troubleshooting so as to ensure accurate pH measurements for the organelle of interest. Additionally, appropriate lasers, filter sets, and detectors are necessary for exciting and detecting the emissions of ratiometric pHluorin (Bencina, 2013).

**Figure 3:**
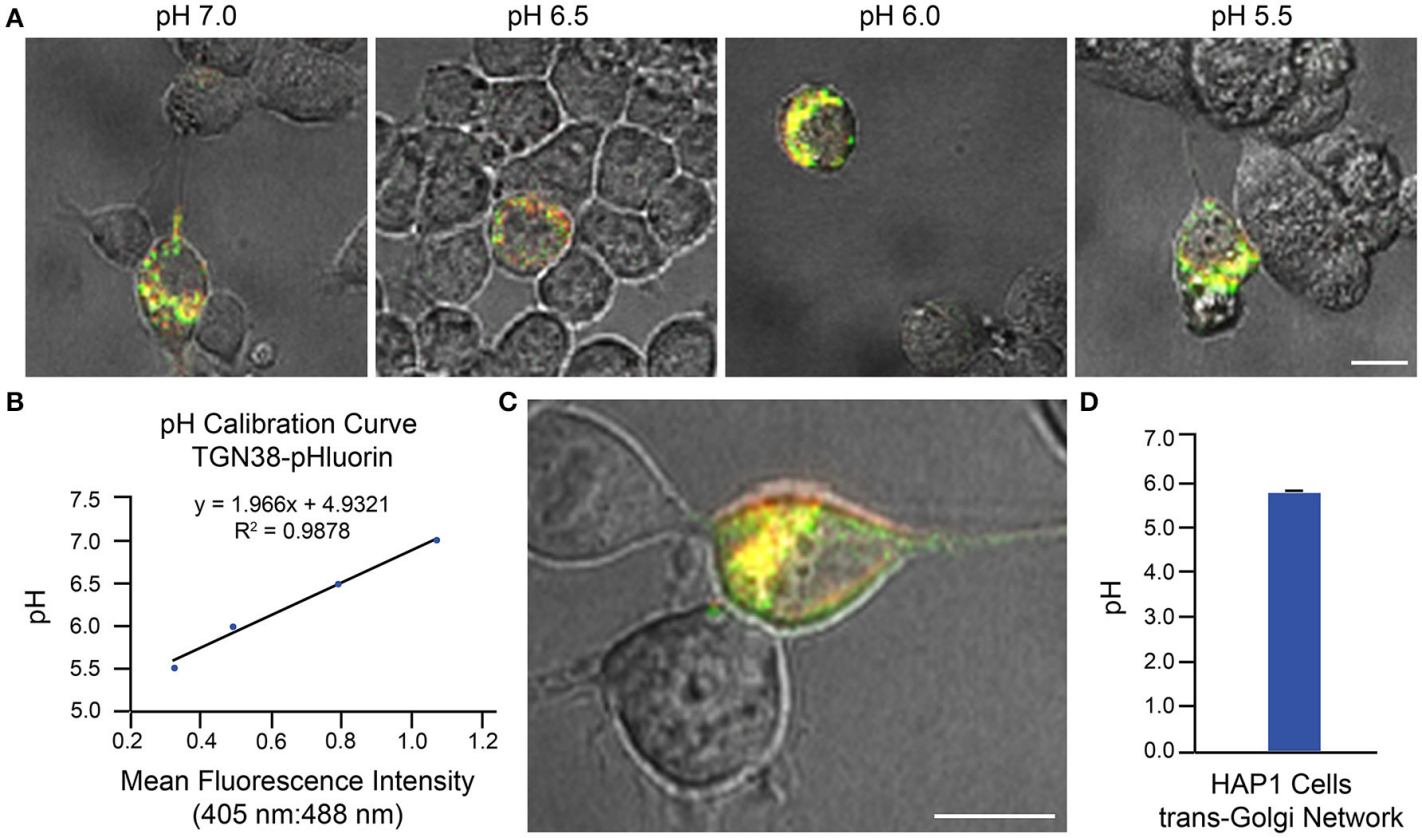
Determination of Golgi pH in HAP1 cells based on use of TGN38-pHluorin and ratiometric fluorescence microscopy. HAP1 cells were transfected with a plasmid encoding for TGN38-pHluorin and subsequently analyzed 24 h post-transfection using ratiometric fluorescence microscopy. Emissions were collected at 530 nm for excitations at both 405 and 488 nm. For images, the collected emissions are color coded red for excitation at 405 nm and green for excitation at 488 nm. **(A)** Fluorescence microscopy images of HAP1 cells expressing TGN38-pHluorin and incubated in pH calibration curve buffers of the indicated pH-values. Scale bar, 10 μm. **(B)** Graph of the pH calibration curve generated based on fluorescence intensity measurements of regions of interest identified in HAP1 cells such as those shown in **(A)**. The pH calibration curve was generated in parallel with acquiring experimental data. **(C)** Representative fluorescence microscopy image of a HAP1 cell expressing TGN38-pHluorin used for collecting experimental data. Scale bar, 10 μm. **(D)** Graph of the average TGN pH in HAP1 cells based on analysis of cells expressing TGN38-pHluorin using ratiometric fluorescence microscopy (*n* = 26). Fluorescence intensity ratios were converted to pH-values by fitting of data to the pH calibration curve shown in **(B)**. The average pH of the TGN for HAP1 cells was calculated at 5.74 ± 0.04. Data are presented as the average ± SEM.

**Figure 4:**
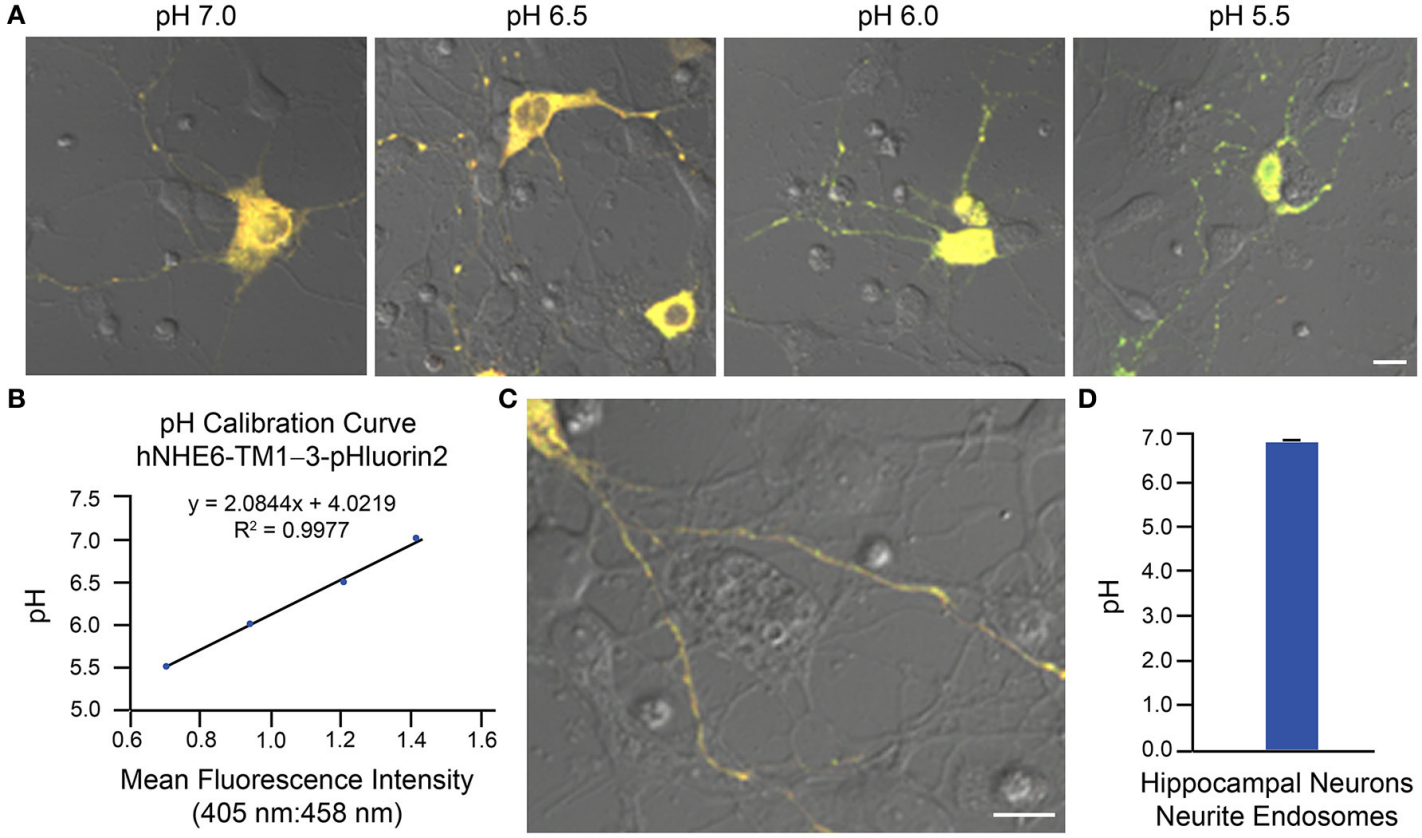
Determination of endosomal pH in neurites of mouse primary hippocampal neurons based on use of ratiometric pHluorin-tagged NHE6 and ratiometric fluorescence microscopy. Mouse primary hippocampal neurons were transfected with a plasmid encoding for hNHE6-TM1–3-pHluorin2 at 2 DIV and subsequently analyzed 24 h post-transfection using ratiometric fluorescence microscopy. Emissions were collected at 500–550 nm for excitations at both 405 and 458 nm. For images, the collected emissions are color coded red for excitation at 405 nm and green for excitation at 458 nm. **(A)** Fluorescence microscopy images of mouse primary hippocampal neurons expressing hNHE6-TM1–3-pHluorin2 and incubated in pH calibration curve buffers of the indicated pH-values. Scale bar, 10 μm. **(B)** Graph of the pH calibration curve generated based on whole-cell fluorescence intensity measurements from neurons such as those shown in **(A)**. The pH calibration curve was generated in parallel with acquiring experimental data. **(C)** Representative fluorescence microscopy image of a mouse primary hippocampal neuron expressing hNHE6-TM1–3-pHluorin2 used for collecting experimental data. Scale bar, 10 μm. **(D)** Graph of the average pH of endosomes in neurites of mouse primary hippocampal neurons based on analysis of neurons expressing hNHE6-TM1–3-pHluorin2 using ratiometric fluorescence microscopy (*n* = 239). Fluorescence intensity ratios were converted to pH-values by fitting of data to the pH calibration curve shown in **(B)**. The average pH of endosomes in neurites of mouse primary hippocampal neurons was calculated at 6.82 ± 0.03. Data are presented as the average ± SEM.

Two examples of implementation of the above protocol for measurement of intraorganellar pH based on use of ratiometric pHluorin-tagged proteins are provided: (1) measurement of trans-Golgi network (TGN) luminal pH in HAP1 cells and (2) measurement of endosomal pH in mouse primary hippocampal neurons. In Figure 3, results are shown for HAP1 cells expressing the TGN-localized protein TGN38-pHluorin (Machen et al., 2003; Maeda et al., 2008). Generation of the pH calibration curve is shown in Figures 3A,B and measurement of TGN pH is shown in Figures 3C,D. Based on this example, the average luminal pH of the TGN for HAP1 cells was calculated at 5.74 ± 0.04. In Figure 4, results are shown for mouse primary hippocampal neurons expressing a plasmid encoding for transmembrane domains 1–3 of human NHE6 fused to pHluorin2 (hNHE6-TM1–3-pHluorin2); pHluorin2 is an enhanced variant of ratiometric pHluorin that exhibits increased fluorescence, in comparison to pHluorin (Mahon, 2011). NHE6 localizes to endosomes where, as a passive Na^+^/H^+^ exchanger that allows H^+^ ions to “leak” out of endosomes, it plays a role in regulating endosome acidification (Ohgaki et al., 2011; Ouyang et al., 2013). Mouse primary hippocampal neurons were transfected at 2 DIV; they were analyzed 24 h post-transfection, upon confirming expression of fluorescent protein. Generation of the pH calibration curve, which was based on whole-cell fluorescence, is shown in Figures 4A,B. For measurement of endosomal pH, regions of interest were limited to neurites (Figures 4C,D). Based on this example, the average pH of endosomes in neurites of mouse primary hippocampal neurons was calculated at 6.82 ± 0.03.

### Measurement of the fluorescent dye LysoSensor™

The LysoSensor™ family member LysoSensor™ Yellow/Blue DND-160 provides a means for ratiometric measurement of intraorganellar pH through use of dual-wavelength fluorescence-based analysis. It is limited to use in living cells, where the fluorescent dye produces yellow fluorescence in acidic environments and blue fluorescence in neutral environments. As for other LysoSensor™ family members, LysoSensor™ Yellow/Blue DND-160 is a weak base and thus can have an alkalinizing effect over time on the organelles to which it partitions (Life Technologies, 2013; Guha et al., 2014). As such, use of this reagent requires one to be rapid and efficient in performing experimental procedures so as to ensure accurate results are obtained. Also, when using a microplate reader as a method of analysis, caution should be exhibited in interpreting results, as aberrant extremely bright signal such as from dead cells or debris and extracellular fluorescence from the dye may contribute to or even dominate the signal.

**Figure 5:**
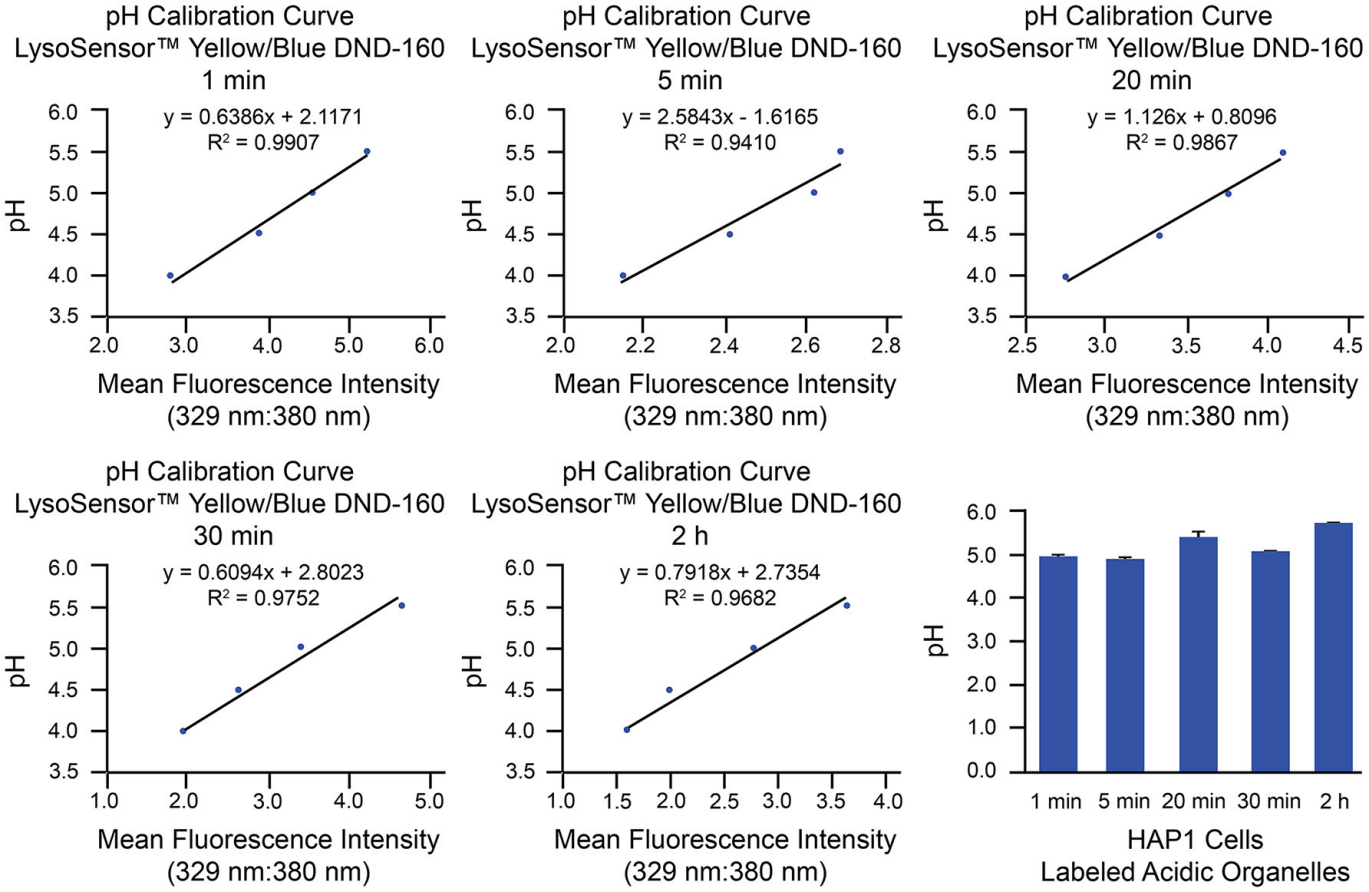
Measurement of intraorganellar pH in HAP1 cells using the ratiometric dye LysoSensor™ Yellow/Blue DND-160 and a microplate reader. HAP1 cells were treated as described in the stepwise procedures with the caveat that, to determine the effects of long-term treatment, cells were incubated with LysoSensor™ Yellow/Blue DND-160 (1 μM) over a time course spanning 1 min to 2 h in generating the pH calibration curves and in acquiring experimental data. Cells were subsequently analyzed in triplicate using a microplate reader and SoftMax® Pro V5 software to determine fluorescence intensity ratios. Emissions were collected at 440 and 540 nm for excitations at 329 and 380 nm, respectively. Graphs are shown of the pH calibration curves generated based on fluorescence intensity measurements of HAP1 cells incubated with pH calibration curve buffers of the indicated pH-values and analyzed using a microplate reader. The pH calibration curves were generated in parallel with acquiring experimental data. The final graph shows the average pH of all labeled acidic organelles combined at various time points of LysoSensor™ Yellow/Blue DND-160 incubation in HAP1 cells, as determined by using microplate reader-based ratiometric measurement. Results from incubation of HAP1 cells with this weak-base dye over a time course are indicative of the rapid alkalinizing effect LysoSensor™ Yellow/Blue DND-160 can have on intraorganellar pH. Fluorescence intensity ratios were converted to pH-values by fitting of data to the corresponding pH calibration curves. For HAP1 cells, the average intraorganellar pH of all LysoSensor™ Yellow/Blue DND-160-labeled organelles combined was calculated at the following values for the respective time points: 4.97 ± 0.08 (1 min), 4.89 ± 0.08 (5 min), 5.4 ± 0.1 (20 min), 5.15 ± 0.04 (30 min), and 5.72 ± 0.02 (2 h). Data are presented as the average ± SEM.

As an example of implementation of the above protocol for ratiometric measurement of LysoSensor™ Yellow/Blue DND-160, we have used HAP1 cells analyzed by microplate reader. This method of analysis provides a measurement of whole-cell fluorescence reflective of all LysoSensor™ Yellow/Blue DND-160-labeled organelles combined. Analysis of luminal pH of specific populations of acidic organelles can be best achieved through use of the described confocal microscopy-based method, on which we have published with respect to primary cultures of mouse hippocampal neurons (Ouyang et al., 2013). Generation of pH calibration curves and corresponding results from microplate reader-based analyses of HAP1 cells loaded with LysoSensor™ Yellow/Blue DND-160 for specific amounts of time (1 min, 5 min, 20 min, 30 min, and 2 h) are shown in Figure 5. Based on these examples, in HAP1 cells, the average intraorganellar pH of all LysoSensor™ Yellow/Blue DND-160-labeled organelles combined was calculated at the following values for the respective time points: 4.97 ± 0.08 (1 min), 4.89 ± 0.08 (5 min), 5.4 ± 0.1 (20 min), 5.15 ± 0.04 (30 min), and 5.72 ± 0.02 (2 h). Importantly, these results are suggestive of the alkalinizing effect LysoSensor™ Yellow/Blue DND-160 can have on intraorganellar pH over extended incubation periods. As such, experimental designs using relatively shorter treatment times (e.g., 1–5 min) are recommended.

## Discussion

Luminal pH is an important functional feature of intracellular organelles. Acidification of the lumen of organelles such as endosomes, lysosomes, and the Golgi apparatus plays a critical role in fundamental cellular processes (Casey et al., 2010; Huotari and Helenius, 2011). As such, measurement of the luminal pH of these organelles has relevance to both basic research and translational research. At the same time, accurate measurement of intraorganellar pH in living cells can be challenging and may be a limiting hurdle for research in some areas. Herein, we have described and provided examples of implementation of three protocols for measuring the luminal pH of different intracellular organelles, focusing on endosomes, lysosomes, and the Golgi apparatus. In introducing the protocols and anticipated results, we have also noted some of the strengths and weaknesses of each of these methods (for a summary, see Table 4).

**Table 4:** Strengths and weaknesses of different methods for measurement of intraorganellar pH.

	**Strengths**	**Weaknesses**
Ratiometric measurement of fluorescent conjugates of transferrin	Internalization of fluorescent conjugates of transferrin takes advantage of a cell's endogenous endocytic pathway and the ubiquitous need for iron.	Localization of the transferrin fluorescent conjugates to specific endosomal compartments is sensitive to the incubation and imaging times and may spread across different endosomal compartments, making accurate measurements for a single type of endosomal compartment difficult. If conjugated to two separate transferrin molecules (as opposed to a single molecule), the pH-sensitive and pH-insensitive fluorescent conjugates of transferrin may exhibit differences in endocytic uptake and/or sorting and vesicle trafficking, thereby leading to inaccurate fluorescence intensity ratios.
Measurement of intrinsically fluorescent ratiometric pHluorin fusion proteins	Use of genetically encoded, pH-sensitive fusion proteins precludes the need for pre-loading of cells with reagents or artificial staining. Plasmids can be generated relatively easily using standard cloning procedures that encode for ratiometric pHluorin-tagged proteins targeted to the luminal domains of specific organelles, thereby allowing for flexibility and specificity.	For some cells types, efficiency of transfection and/or protein expression levels may pose a limitation. As exogenously expressed proteins, the potential for aberrant localization patterns of ratiometric pHluorin fusion proteins exists and may present a concern.
Measurement of the fluorescent dye LysoSensor™	Internalization of the fluorescent dye takes advantage of the intrinsic accumulation of membrane-permeant weak bases in the lumens of acidic organelles.	LysoSensor™ Yellow/Blue DND-160 can have an alkalinizing effect on the organelles to which it partitions, which may affect the accuracy of results.
Use of confocal microscopy	Spatial and morphological information can be obtained. Visualization and selection of labeled organelles can be performed. Artifacts, apoptotic cells, and background noise can be discarded, making calculations easier and more accurate.	Microscopy may not be optimal for use with semi-adherent or non-adherent cell types. The selection and analysis of specific regions of interest is required, which may be time consuming and potentially limit the number of cells that can be analyzed efficiently. Photobleaching, including with respect to differences in the susceptibility to and rates of for different fluorophores, and/or photolysis may present a concern at higher laser intensities and over longer time periods of imaging.
Use of flow cytometry/FACS or Use of microplate reader	Semi-adherent or non-adherent cells can be analyzed using these methods. Many cells can be analyzed in a single reading. Photobleaching and photolysis are of limited concern. Large populations of cells can be analyzed in a sufficiently rapid manner so as to acquire data relating to acidification kinetics (flow cytometry/FACS). Dead or dying cells can be excluded from analysis (flow cytometry/FACS).	Discrete regions of interest cannot be selected for analysis, including exclusion of signal from extracellular fluorescence. Little to no spatial or morphological information is obtained. A readout of whole-cell fluorescence is provided, resulting in the average intraorganellar pH of all labeled organelles for the analyzed cell population. The signal may be dominated by a few extremely bright, but essentially irrelevant, cells (e.g., dead cells) or dye-labeled debris particles (microplate reader).

The protocols we have presented represent powerful, diverse approaches for determining intraorganellar pH in living cells. In ranging from use of fluorescent conjugates of transferrin to ratiometric pHluorin fusion proteins to a fluorescent dye of the LysoSensor™ family, the protocols provide for various options depending on the experimental question at hand. A key aspect of these protocols is performing procedures for the generation of a pH calibration curve in parallel with performing corresponding procedures for making experimental measurements of intraorganellar pH. For each protocol and for each of these two components, we have provided stepwise procedures for: (1) loading of cells with the fluorescent reagent or transfection of cells with a plasmid encoding for a fluorescent protein, (2) data acquisition, whether it be images, flow cytometry/FACS data, or results from reading of microplates, and (3) data analysis, including plotting of data for generating the pH calibration curve and fitting of data to the pH calibration curve for converting experimental measurements to pH-values. Also, complete stepwise procedures in the context of an alternate method of analysis are indicated for two of the protocols so as to aid in adaptability to particular experimental conditions. Taken together, the protocols have the potential for broad applicability. They might be implemented in addressing research questions more basic in nature, as well as in studies with translational implications.

## Author contributions

LM, QO, and EM designed the experiments. LM, QO, and GW performed the experiments and acquired the data. All authors analyzed the data and discussed the results. All authors contributed to the drafting and/or revision of the manuscript and approved of the final version of the manuscript.

### Conflict of interest statement

The authors declare that the research was conducted in the absence of any commercial or financial relationships that could be construed as a potential conflict of interest.

## Footnotes

^1^In generating the pH calibration curve, it is important to recall that a buffer offers optimal buffering capacity when in a solution of pH-value at or near the p*K*_a_ of the buffer. As such, the pH calibration curve will be most linear over the range of pH-values near the p*K*_a_-values of the buffers used in preparing the pH calibration curve buffers to be used as standards. Also, it is over the linear portion of the curve that the change in fluorescence intensity ratio is greatest (i.e., greatest slope). Combined, the pH calibration curve buffers should span a relatively wide overall range (e.g., pH 4–7), and at least two different buffer systems of appropriate p*K*_a_-values should be used. Also, to the extent possible, it should be ensured that the experimental data (i.e., fluorescence intensity ratios) fall within the linear portion of the curve so as to allow for the best opportunity to detect differences in pH-values if indeed present. Of further note and extending on the above, careful consideration of the dynamic range of the calibration curve is warranted. Collecting a calibration curve for each experimental condition could be helpful. While calibration curves will vary based on specific conditions, these curves should be very similar for like conditions. Calibration curves that vary substantially despite being obtained under similar conditions and using similar probes (i.e., lack consistency) and/or show limited dynamic range should raise caution regarding quality of data and warrant further exploration within a given experiment.

^2^For optimal results, cells should be maintained in a CO_2_ chamber held at 37°C or at minimum placed on a heated stage held at 37°C during imaging. If cells are imaged in a CO_2_ chamber, it is important to use imaging medium that contains bicarbonate at an appropriate concentration for the percent CO_2_ so as to ensure maintenance of the medium at a physiological pH. Conversely, if cells are imaged in air, it is important to use imaging medium lacking bicarbonate, as escape of CO_2_ into the atmosphere would result in alkalinization of the medium. Alternatively, imaging medium using a buffering system independent of bicarbonate, such as HEPES buffer, can be used under both imaging conditions. See Dunn et al. (1994) for an example in which endosomal pH was determined in the context of cells imaged in a CO_2_ chamber vs. in air in the presence or absence of bicarbonate buffer, respectively.

^3^Fluorescently labeled organelles appropriate for analysis may be localized at different focal planes within a cell population or even within a single cell. As such, to maximize the number of organelles that can be analyzed in a given image, it may be of benefit to acquire z-sections of images (e.g., 0.5–1.0 μm steps) and generate stacked images or maximum projection profiles for analysis.

^4^The time allowed for transferrin loading, as well as the time period over which images are acquired, will determine the endosomal populations that are labeled. Furthermore, as vesicle trafficking and endosome maturation are dynamic processes, with the kinetics with respect to transferrin trafficking differing for different cell types (Mayle et al., 2012), multiple endosomal populations are likely to be labeled in a given image. This feature should be taken into consideration when determining regions of interest for analysis and when interpreting experimental results. Also, depending on cell type and the endosomal population of main interest for analysis, the indicated time periods for transferrin loading and imaging may need to be adjusted. If using the confocal microscopy-based method of analysis, an option for ensuring labeling of very early endosomes generated essentially immediately following formation of endocytic vesicles is to include the fluorescent conjugates of transferrin in the medium during imaging (Dunn et al., 1994).

^5^In using LysoSensor™ Yellow/Blue DND-160, care should be taken to use a working concentration of dye that is as low as possible while still providing for sufficient organellar staining so as to reduce the potential for artifacts from sample overloading. Also, as weak bases, the LysoSensor™ family of fluorescent dyes can exhibit an alkalinizing effect. As such, longer incubation times with these fluorescent dyes can induce an increase in the pH of the labeled acidic organelles. A recommended concentration is at least 1 μM, but may be from 2 to 5 μM, and a suggested time period for incubation is 1–5 min (Guha et al., 2012, 2014; Life Technologies, 2013; Lu et al., 2013; Coffey et al., 2014). Optimal conditions should, however, be determined for the cell type under investigation.
