# Supplementary material for: Live-cell Microscopy and Fluorescence-based Measurement of Luminal pH in Intracellular Organelles
Source: Front Cell Dev Biol. 2017 Aug 21;5:71. doi: 10.3389/fcell.2017.00071 (PMC5566985; doi:10.3389/fcell.2017.00071)
Supplement: Supplementary file 1 [file Table1.DOCX]

**Table S1. Primers for generating the plasmid encoding for hNHE6-TM1**−**3-pHluorin2.**

| **Primer Name** | **Primer Sequence 5’-3’** |
| --- | --- |
| NHE6-T1-F | GAATTCGTCGAGAGAGGATGG |
| NHE6-T1-3R-BamHI | CTTGGATCCACTTCACAGCTCAGGGTCAC |
| T1-3-pHluorin2F-BamHI | CCAGGATCCGGTGAGCAAGGGCGAGGAG |
| T1-3-pHluorin2R | GGATCACTTGTACAGCTCGTCCATGCCGTG |
